# Outcome of 5-year follow-up in men with negative findings on initial biparametric MRI

**DOI:** 10.1016/j.heliyon.2021.e08325

**Published:** 2021-11-06

**Authors:** Karen-Cecilie Kortenbach, Lars Boesen, Vibeke Løgager, Henrik S. Thomsen

**Affiliations:** Herlev Gentofte University Hospital, Department of Radiology, Borgmester Ib Juuls vej 17, DK-2730 Herlev, Denmark

**Keywords:** Biparametric MRI, Multiparametric MRI, Prostate cancer

## Abstract

**Background:**

We assessed the 5-year risk of being diagnosed with significant prostate cancer following a low-suspicion biparametric magnetic resonance imaging result.

**Methods:**

The study population was derived from a prospective database used to assess the diagnostic accuracy of biparametric magnetic resonance imaging for significant prostate cancer detection in 1020 biopsy-naïve men. Significant prostate cancer was defined as any core with Gleason grade group ≥3 or a maximum cancerous core length greater than 50% of Gleason grade group 2. A secondary definition of significant prostate cancer was also included: any core with prostate cancer Gleason grade group ≥2. Of the 1020 men, 305 had a low-suspicion biparametric magnetic resonance imaging result (Prostate Imaging Reporting and Data System score of 1 or 2) but four men were excluded from follow-up. Thus, the final study population consisted of 301 men, who were clinically followed-up from inclusion (November 2015 to June 2017) until 1 June 2021.

**Findings:**

Overall, 1·7% (5/301) of the study population had significant prostate cancer diagnosed within 5 years (median 1480 days, Interquartile Range (1587–1382)) of their low-suspicion result and corresponding set of biopsies. When the secondary definition of significant prostate cancer was applied, this increased to 5% (15/301) of the study population.

**Interpretation:**

The 5-year risk of being diagnosed with significant prostate cancer after a prebiopsy low-suspicion prebiopsy biparametric magnetic resonance imaging result was 1·7%.

## 1 Introduction

Previously, when a man was suspected of having prostate cancer (PCa) he was offered a transrectal ultrasound-guided prostate biopsy (TRUS-bx). This approach contributed to increased PCa detection but also led to men without PCa undergoing unnecessary biopsies and to a substantial increase in men diagnosed and treated for clinically insignificant (ins) PCa [1]. Furthermore, biopsies are invasive and can lead to complications such as severe infection and rectal bleeding [2, 3, 4]. A better way to diagnose PCa was needed. Multiparametric (mp) magnetic resonance imaging (MRI) followed by MRI targeted biopsies emerged as an alternative diagnostic tool for improved detection of significant (s) PCa compared with TRUS-bx alone [5, 6, 7, 8, 9]. This strategy avoids unnecessary biopsies, with their inherent complications, improves the detection rate of sPCa, and reduces detection of insPCa [5, 6, 7, 8, 9]. Thus, current guidelines now recommend mpMRI prior to biopsy [10, 11]. However, multiple studies have shown that abbreviated biparametric (bp) MRI has equivalent sPCa detection rates and may be more cost-effective than mpMRI [8, 12, 13, 14, 15, 16, 17, 18, 19]. The Biparametric MRI for Detection Of prostate Cancer (BIDOC) study showed that a low-suspicion bpMRI result had a high negative predictive value in ruling out sPCa and may be used to exclude the presence of aggressive disease thereby avoiding unnecessary biopsies [19]. Furthermore, the BIDOC study showed that men with low-suspicion bpMRI results could safely avoid biopsies, as most had either benign findings on confirmatory biopsies or insPCa that could be managed with expectance [19]. However, PCa often develops slowly [20]. Therefore, long-term follow-up is needed to evaluate the overall consequences of utilising bpMRI as a triage test to identify biopsy-naïve men with clinical suspicion of PCa who might safely avoid invasive biopsies. This study assessed the 5-year risk of being diagnosed with sPCa following a low-suspicion bpMRI result.

## 2 Methods

**Figure 1:**
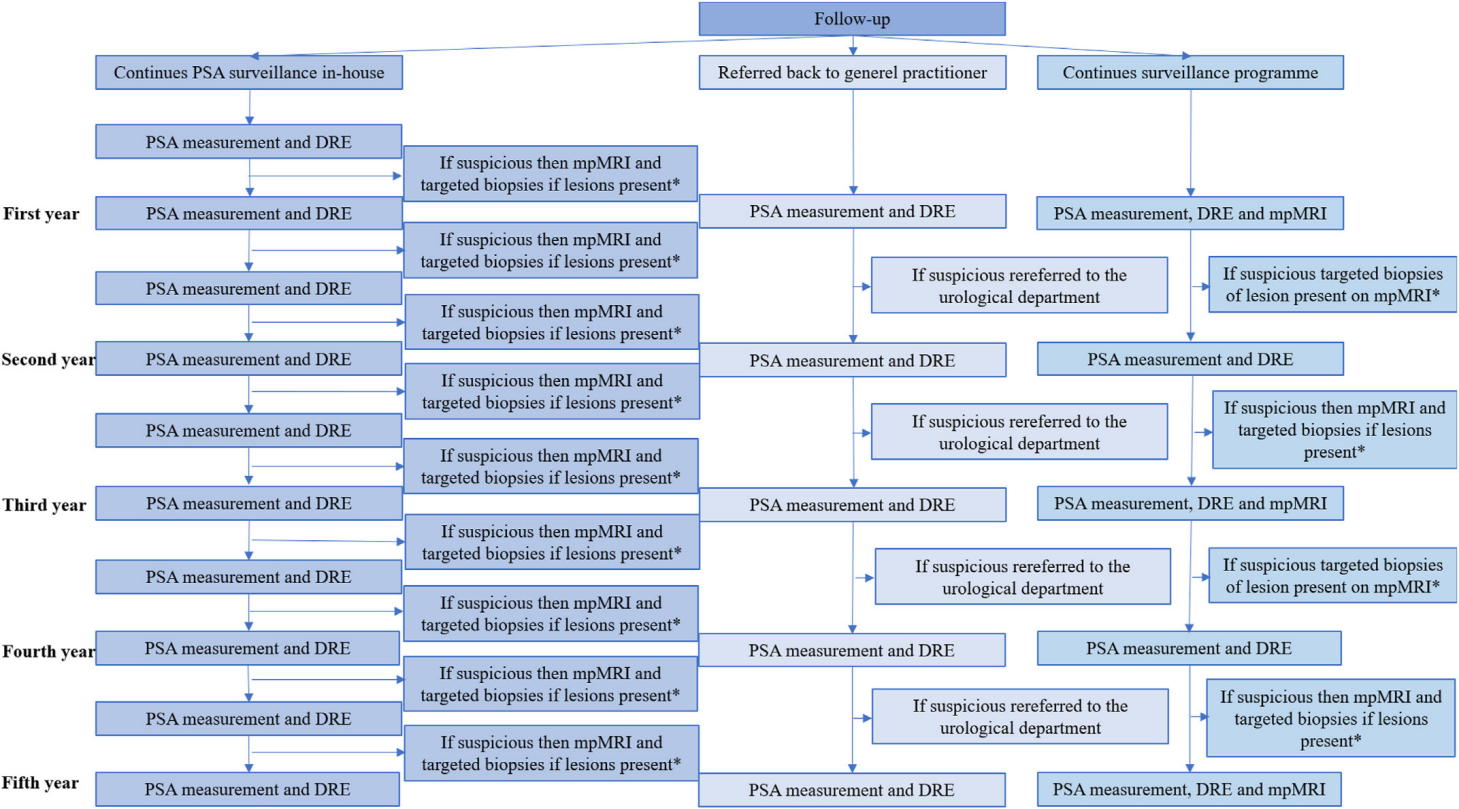
Follow-up structure. ∗Depending on the biopsy result the man either continued his path or had treatment accordingly. Abbreviations: PCa, prostate cancer; PSA, prostate-specific-antigen; DRE, digital rectal examination; mpMRI, multiparametric magnetic resonance imaging.

The study population was derived from the prospective database that the BIDOC study used to assess the diagnostic accuracy of bpMRI for PCa detection in 1020 biopsy-naïve men [19]. The BIDOC study was approved by the Local Committee for Health Research Ethics and the Danish Data Protection Agency and participants provided written informed consent [19]. The BIDOC database included 1020 biopsy-naïve men with clinical suspicion of PCa who underwent bpMRI followed by TRUS-bx in all men and targeted biopsies of any lesion with a Prostate Imaging Reporting and Data System (PI-RADS) score ≥3 during the period November 2015 to June 2017. Of these, 305 men had a low-suspicion bpMRI result (PI-RADS score 1 or 2). Which almost certainly indicates the absence of PCa (very low likelihood). Four men were excluded from this follow-up study because they refused to undergo any further examinations or surveillance. Thus, the final study population consisted of 301 men, who were clinically followed-up from inclusion in the BIDOC study (November 2015 to June 2017) until 1 June 2021 (Figure 1) [19]. Because, the BIDOC study initially did not include a pre-specified follow-up protocol, the follow-up intervals was not exactly the same for included patient but is described in Figure 1. The men were clinically followed, and the last follow-up was defined as either last visit at the urological department or last PSA measurement at their general practitioner (GP).

Men with a low-suspicion bpMRI result and benign biopsies were either referred back to their GP, underwent treatment for lower urinary tract symptoms or continued prostate-specific-antigen (PSA) surveillance in-house. In-house PSA surveillance consisted of PSA measurements plus digital rectal examinations (DREs) by a urologist every three to six months, and mpMRI with repeated biopsies was performed at the discretion of the treating urologist if progression was suspected due to rising PSA and/or suspicious DRE giving clinical suspicion of missed or newly developed sPCa. Men who were referred back to their GPs were also advised to undergo PSA monitoring plus DREs every six to twelve months. These men had individualised thresholds for rereferral to the urological department with clinical suspicion of new or missed sPCa.

Men with a low-suspicion bpMRI result but a PCa positive confirmatory biopsy either continued in surveillance programmes (i.e., active surveillance or watchful waiting) or progressed directly to remedial treatment (i.e., radical prostatectomy or external beam radiation therapy). Men in active surveillance had remedial treatment if their urologist deemed this necessary, based on DRE, PSA measurement, MRI and biopsy results.

All men who were rereferred to the urological department, based on their individualised risk-thresholds, were reassessed by the treating urologist for persistent clinical suspicion. This included men with suspicious DRE results and/or rising PSA levels. A confirmatory mpMRI was performed in men with persistent clinical suspicion of sPCa followed by combined (i.e., standard plus targeted) biopsies of mpMRI positive men. If clinical suspicion of sPCa was deferred after these men were assessed at the department of urology, PSA surveillance was continued in-house or by the GP.

### 2.1 Magnetic resonance imaging

All MRI examinations were performed using a 3-T MRI magnet (Ingenia version 5.3.1; Philips Healthcare, Best, the Netherlands) with a 16-channel surface coil and a built-in table coil (Philips Healthcare) positioned over the pelvis. The bpMRI protocol included axial T2-weighted and diffusion-weighted imaging with reconstructions of the corresponding apparent diffusion coefficient maps and image acquisition times of approximately 15 min. Detailed descriptions of the mpMRI and bpMRI protocols are provided in Table 1. Further details are located in supplementary material Table 1. All bpMRIs were reviewed by the same prostate MRI physician (>8 years of experience) who registered and scored any suspicious lesions on a 5-point scale according to their likelihood of being sPCa (1, highly unlikely; 2, unlikely; 3, equivocal; 4, likely; and 5, highly likely) using the PI-RADS version 2.0 criteria [21]. The bpMRI protocol does not include dynamic contrast-enhanced imaging, scoring of lesions in the peripheral zone relied solely on diffusion-weighted image findings (dominant sequence), and an equivocal score of 3 was not potentially upgraded to a score of 4 due to lack of positive dynamic contrast-enhanced findings. A modified PI-RADS suspicion score of 2 or lower was perceived as a low-suspicion or negative bpMRI scan result. Men with PI-RADS 2 scores or lower did not undergo targeted biopsies.

**Table 1:** MpMRI and bpMRI protocols.

	TR (ms)	TE (ms)	Fov (mm × mm)	Matrix	b values	NSA	Slice (mm)	Scan time (min:s)	Temp res (min:s)
**mpMRI**∗									
T2 sag	3000	90	160 × 198	268 × 326		2	3	06:06	
T2 ax	4000	90	180 × 180	400 × 400		1	3	09:19	
DWI ax	13743	71	180 × 180	84 × 80	0; 100; 800; 2000	2	4	06:33	
T2 cor	3504	90	190 × 190	316 × 312		1	3	04:16	
DCE∗∗	10	5	180 × 158	256 × 221		1	5	03:03	00:15
**bpMRI**									
T2 sag (scout)	3	1·65	270 × 270	180 × 180		2	3	00:29	
T2 ax	3475	90	180 × 180	400 × 400		1	3	08:14	
DWI ax	10000	71	180 × 180	84 × 80	0; 100; 800; 2000	2	4	06:30	

Abbreviations: mpMRI, multiparametric magnetic resonance imaging; bp, biparametric (MRI); TR, repetition time; TE, echo time; Fov, field of view; NSA, number of signal averages; Temp res, temporal resolution; sag, sagittal; ax, axial; DWI, diffusion-weighted images; cor, coronal; DCE, dynamic contrast-enhanced; ms, milliseconds.

∗ Administered if tolerated (1 mL hyoscine butylbromid [20 mg/mL Buscopan injection fluid; Boehringer Ingelheim GmbH, Ingelheim am Rhein, Germany] and 1 mL glucagon [1 mg/mL GlucaGen; Novo Nordisk A/S, Bagsværd, Denmark]).

∗∗ Administered 0·2 mL/kg gadoteric acid [15–20 mL 279·3 mg/mL Dotarem injection fluid; Guerbet B. P., Villepinte, France].

### 2.2 Histopathological evaluation

All biopsy samples were reviewed by the same genitourinary pathologist (>16 years of experience). The location, the Gleason Score and the percentage of cancerous tissue per core were determined based on the International Society of Urological Pathology 2005 consensus guidelines for each PCa-positive biopsy core [22]. In addition, men with tumour-containing biopsies were assigned to a Gleason grade group (GG) in accordance with the International Society of Urological Pathology 2014 consensus guidelines [23]. The definition of sPCa was any core with GG ≥ 3 PCa or a maximum cancerous core length greater than 50% of GG 2 PCa. This primary definition of sPCa was the same as that used in the BIDOC study [19]. Our secondary definition of sPCa was any core with PCa GG ≥ 2.

### 2.3 Statistical analysis

Patient characteristics is presented using descriptive statistics, where means and standard deviation is used to describe continuous variables (e.g., age, PSA level, PSA density, and prostate volume). The normality of the data is assessed using the Shapiro–Wilk method. RStudio software (ver. 1·4·1103; RStudio, Inc., Boston, MA, USA) was used to perform statistical analysis and a *p*-value < 0·05 is considered statistically significant [24].

## 3 Results

**Figure 2:**
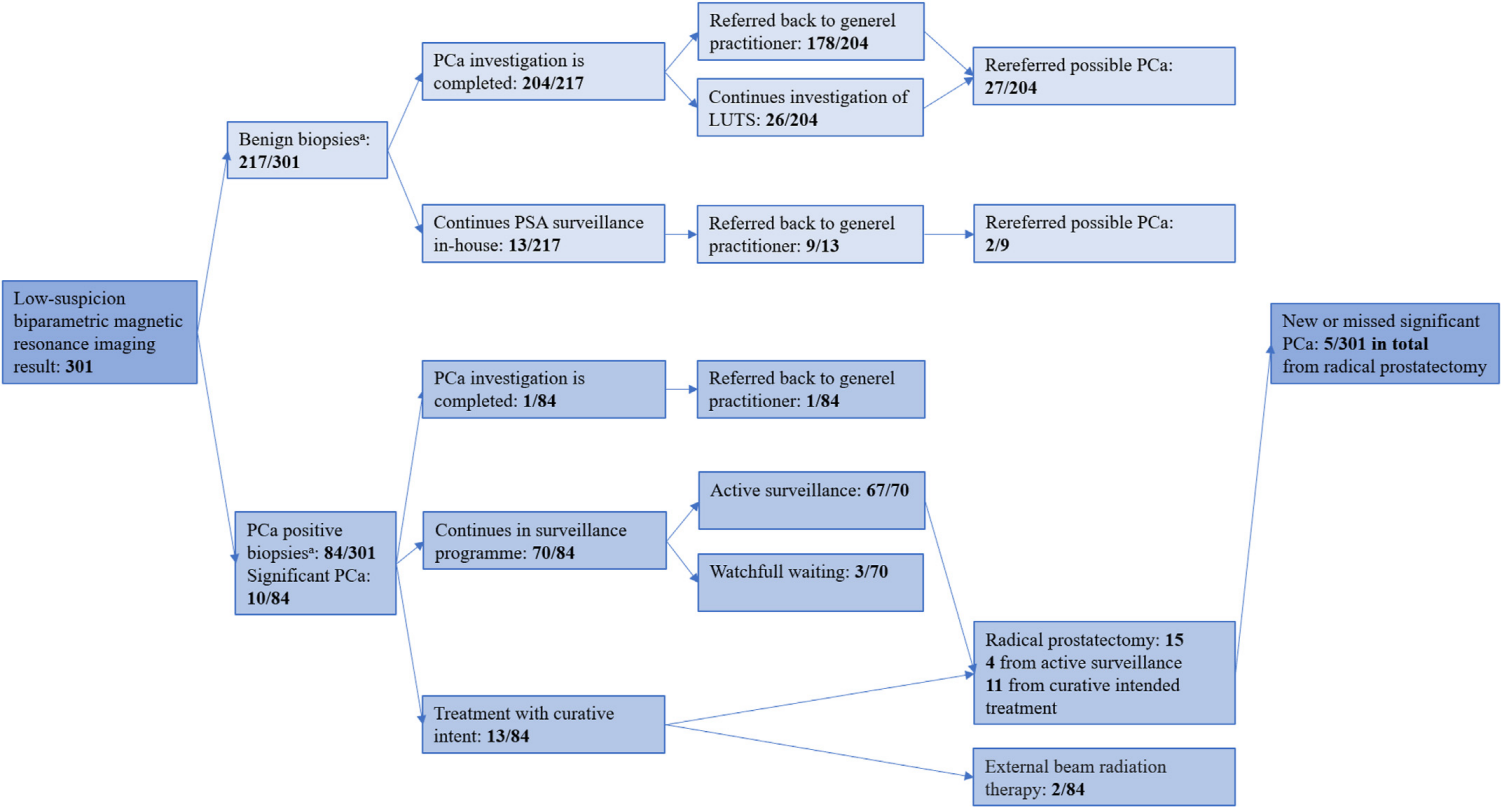
Outcome with primary definition of significant prostate cancer. ^a^: Primary definition of significant prostate cancer: any core with high-grade prostate cancer (GG ≥ 3) or a maximum cancerous core length greater than 50% of GG 2 prostate cancer. Abbreviations: PCa, prostate cancer; low-suspicion biparametric magnetic resonance imaging result, only PI-RADS score <3; GG, Gleason grade group; PSA, prostate-specific-antigen; LUTS, lower urinary tract symptoms.

Demographic and baseline characteristics of the study population are shown in Table 2. Further details on follow-up events over time for each man and the follow-up length for each man is depicted and located in supplementary material Figures 1 and 2. Based on the primary definition of sPCa, 1·7% (5/301) of the men had sPCa diagnosed within 5 years of their low-suspicion bpMRI result and corresponding set of biopsies (Figure 2). Overall, sPCa was detected in 5% (15/301) on either corresponding biopsies at inclusion (n = 10) or during follow-up (n = 5) (Table 2, Figure 2). However, no man with low-suspicion bpMRI and benign biopsies at inclusion (0/217) had sPCa detected during follow-up (Figure 2). Of men with low-suspicion bpMRI results and corresponding PCa positive biopsies at inclusion (84/301), a total of 6% (5/84) had sPCa (Figure 2).

**Table 2:** Demographic and baseline characteristics.

Clinical Characteristic	Population (*n* = 301)	Benign biopsies (*n* = 217)	PCa positive biopsies (*n* = 84)	*p*-value
Age in years, mean (SD)	64 (±7)	64 (±7)	64 (±6)	0·18
PSA, mean (SD), ng/mL	7 (±4)	7 (±4)	7 (±3)	0·33
Prostate volume, mean (SD), cm^3^	73 (±36)	77 (±36)	61 (±32)	0·01
PSA density, mean (SD), ng/mL/cm^3^	0·1 (±0·1)	0·1 (±0·1)	0·1 (±0·1)	0·01
Months from bpMRI to end of follow-up, median (IQR)	1480 (1587–1382)	1643 (1750–1543)	1552 (1622–1453)	0·06
**cTDRE stage, number (%)**				
Non-palpable tumours				
Tx	96 (31·9%)	77 (35·5%)	19 (22·6%)	
T1c	175 (58·1%)	120 (55·3%)	55 (65·5%)	
Palpable tumours				
T2a	21 (7%)	15 (6·9%)	6 (7·1%)	
T2b	5 (1·7%)	3 (1·4%)	2 (2·4%)	
T2c	4 (1·3%)	2 (0·9%)	2 (2·4%)	
**GG, number (%)**				
GG 0	217 (72·1%)	217 (100%)		
GG 1	64 (21·3%)		64 (76·2%)	
GG 2 with MCCL <50%	10 (3·3%)		10 (11·9%)	
GG 2 with MCCL ≥50%	3 (1%)		3 (3·6%)∗	
GG 3	4 (1·3%)		4 (4·8%)	
GG 4	2 (0·7%)		2 (2·4%)	
GG 5	1 (0·3%)		1 (1·2%)	

Abbreviations: PCa, prostate cancer; bpMRI, biparametric magnetic resonance imaging; cTDRE, tumour stage determined by digital rectal examination; MCCL, maximum cancer core length; SD, standard deviation; IQR, Interquartile Range; PSA, prostate-specific-antigen; GG, Gleason grade group.

SI conversion factor: To convert PSA to micrograms per litre, multiply by 1.0.

∗ In two cases a mismatch between biopsy results and clinical evaluation by the urologist were suspected and repeat biopsies were done.

**Figure 3:**
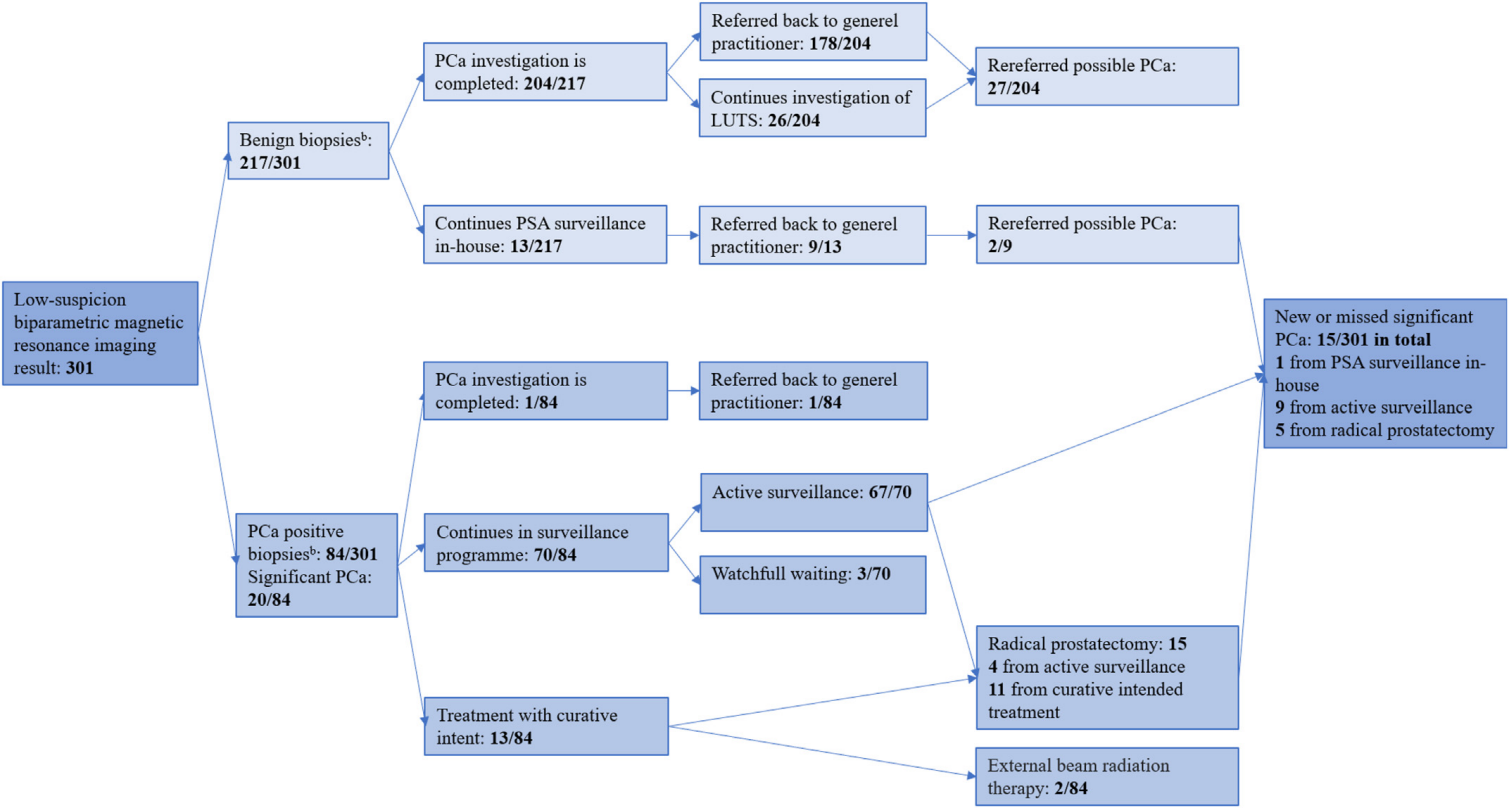
Outcome with secondary definition of significant prostate cancer. ^b^: Secondary definition of significant prostate cancer: any core with prostate cancer Gleason grade group ≥2. Abbreviations: PCa, prostate cancer; low-suspicion biparametric magnetic resonance imaging result, only PI-RADS score <3; GG, Gleason grade group; PSA, prostate-specific-antigen; LUTS, lower urinary tract symptoms.

Based on the secondary definition of sPCa, 5% (15/301) of men with low-suspicion bpMRI results had sPCa diagnosed within 5 years (Figure 3) and the overall number is 8·3% (25/301) because another n = 10 men were diagnosed at inclusion on corresponding set of biopsies (Table 2, Figure 3). Of the men with benign corresponding biopsies, one man 0·5% (1/217) had a new or missed sPCa, according to the secondary definition (Figure 3). This man continued in-house PSA surveillance and had an mpMRI, which revealed a PI-RADS 4 lesion that was subsequently targeted with biopsies. The reader was able to locate this lesion on the bpMRI retrospectively. The biopsies revealed a GG 2 lesion with a maximum cancerous core of 40%. The man was reallocated from in-house PSA monitoring and enrolled in active surveillance, where he remained at the most recent follow-up visit. Of the men with low-suspicion bpMRI results and PCa positive biopsies 16·7% (14/84) had sPCa (Figure 3).

Among the men with benign biopsies, 13% ((27 + 2)/217) were rereferred to the urological department with new suspicion of PCa (Figures 2 and 3). Here they were re-assessed by a urologist and 45% (13/29) continued to exhibit suspicious PSA levels or DREs causing reasonable suspicion of sPCa, and a new mpMRI was recommended. Of these, 10 men consented to having an mpMRI and one of these men had a PI-RADS 4 lesion. The man with the PI-RADS 4 lesion and three others who refused mpMRI had new TRUS-bx and targeted biopsies of the PI-RADS 4 lesion. None of these men exhibited any malignancies in their biopsies but one of the men who had refused to have an mpMRI exhibited chronic inflammation as a possible explanation for persistent elevated PSA levels.

## 4 Discussion

We found that a prebiopsy low-suspicion bpMRI result lowers the risk of sPCa within 5 years to below 2%. By including the number of sPCas detected by the corresponding biopsies at inclusion the number increased to 5% for the primary definition and 8% for the secondary definition. Thus, bpMRI could preferably be combined with other diagnostic tools such as PSA-density, age, and tumour stage information for clinical management. Boesen *et al.* found that these parameters together with bpMRI improved risk stratification for sPCa in biopsy-naïve men and could be used for clinical decision-making and counselling men prior to prostate biopsies [25]. None of the men in our study with low-suspicion bpMRI results and benign biopsies had sPCa detected over a median follow-up period of 5 years, although 13% of these men were rereferred by their GPs. Consequently, if we only do a bpMRI and no biopsies we might miss those 5%. All sPCas were discovered during the first round of investigation in our study which indicates that our safety net of surveillance programmes worked. Thus, there have to be either a safety net or bpMRI needs to be combined with other diagnostic tools if we are to forego the biopsies after a low-suspicion bpMRI.

The key concern in clinical practice is to detect and rule out significant disease while avoiding unnecessary biopsies. MpMRI can be used to meet these objectives but imposes a major financial burden on healthcare systems [26, 27], whereas widespread implementation of bpMRI would represent a more cost-effective approach. A meta-analysis by Bass *et al.* suggested that bpMRI compared favourably with mpMRI and could be used as a prebiopsy triage-test to identify those biopsy-naïve men with clinical suspicion of PCa who might safely avoid invasive biopsies, without sacrificing diagnostic accuracy [16]. Recently, a nomogram for predicting prostate biopsy outcomes based on bpMRI results was developed and internally validated; a prospective multi-institutional nomogram was also developed [17, 25]. None of these studies were supported by long-term analyses; however, our study also indicates that bpMRI may be used as a prebiopsy triage-test to minimise missed sPCa at diagnosis but it cannot stand alone.

Our study had some limitations. This study is a retrospective study and was not designed before the BIDOC study was conducted. This is a limitation because a strict follow-up protocol was not pre-specified, and therefore, the men did not undergo the same intervals between clinical controls including PSA measurements, MRIs and eventually re-biopsies. Another limitation is the number of subjects included. It is quite substantial but due to small prevalence of sPCa, more men would be needed for better statistics. We would need an even larger sample to provide results with higher certainty. Because PCa develops slowly, increasing the follow-up time beyond 5 years may be beneficial [20]. Our study was performed at a single centre with one dedicated MRI physician reading the bpMRIs and two highly experienced TRUS operators performing biopsies. Less experienced readers and operators might not achieve the same diagnostic yield. Prostatectomy is the gold standard for confirming sPCa is present but only a few of the men in our study had a prostatectomy. Thus, we cannot be sure that all sPCas were detected. However, because all men in this study were followed up for 5 years and all who exhibited new or elevated risk factors were assessed, is it not likely that many sPCas were missed.

In conclusion, the 5-year risk of being diagnosed with sPCa after a prebiopsy low-suspicion bpMRI result is 1·7%. Further studies are needed where bpMRI is combined with other diagnostic tools.

## Declarations

### Author contribution statement

Karen-Cecilie Kortenbach: Conceived and designed the experiments; Analyzed and interpreted the data; Contributed reagents, materials, analysis tools or data; Wrote the paper.

Lars Boesen: Conceived and designed the experiments; Analyzed and interpreted the data; Contributed reagents, materials, analysis tools or data.

Vibeke Løgager: Contributed reagents, materials, analysis tools or data.

Henrik S. Thomsen: Conceived and designed the experiments; Contributed reagents, materials, analysis tools or data.

### Funding statement

This work was supported by the Danish Cancer Society (R269-A15896).

### Data availability statement

Data will be made available on request.

### Declaration of interests statement

The authors declare no conflict of interest.

### Additional information

No additional information is available for this paper.

## Appendix A Supplementary data

sup. Figure 1 sup. Figure 1

sup. Figure 2 sup. Figure 2

sup. Table 1 sup. Table 1
