# Supplementary figures and images for: Outcome of 5-year follow-up in men with negative findings on initial biparametric MRI
Source: Heliyon. 2021 Nov 6;7(11):e08325. doi: 10.1016/j.heliyon.2021.e08325 (PMC8601994; doi:10.1016/j.heliyon.2021.e08325)

**Number of days from diagnostic biopsies to follow-up end for each man**


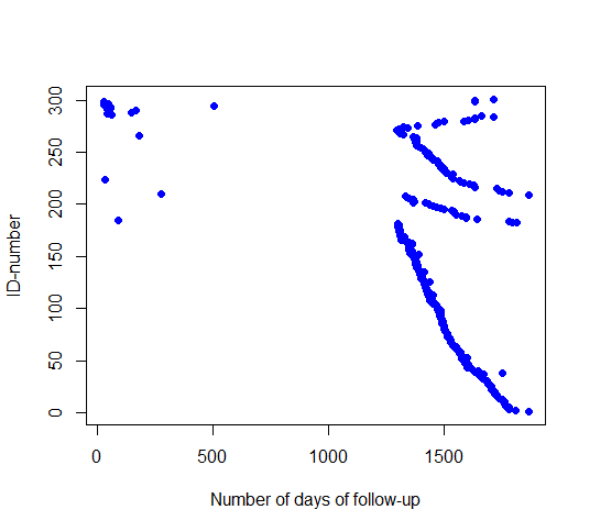

Supplement: sup. Figure 1 [file mmc1.docx]
