# Supplementary material for: Outcome of 5-year follow-up in men with negative findings on initial biparametric MRI
Source: Heliyon. 2021 Nov 6;7(11):e08325. doi: 10.1016/j.heliyon.2021.e08325 (PMC8601994; doi:10.1016/j.heliyon.2021.e08325)
Supplement: sup. Figure 2 [file mmc2.docx]

**Follow-up events over time for each man**


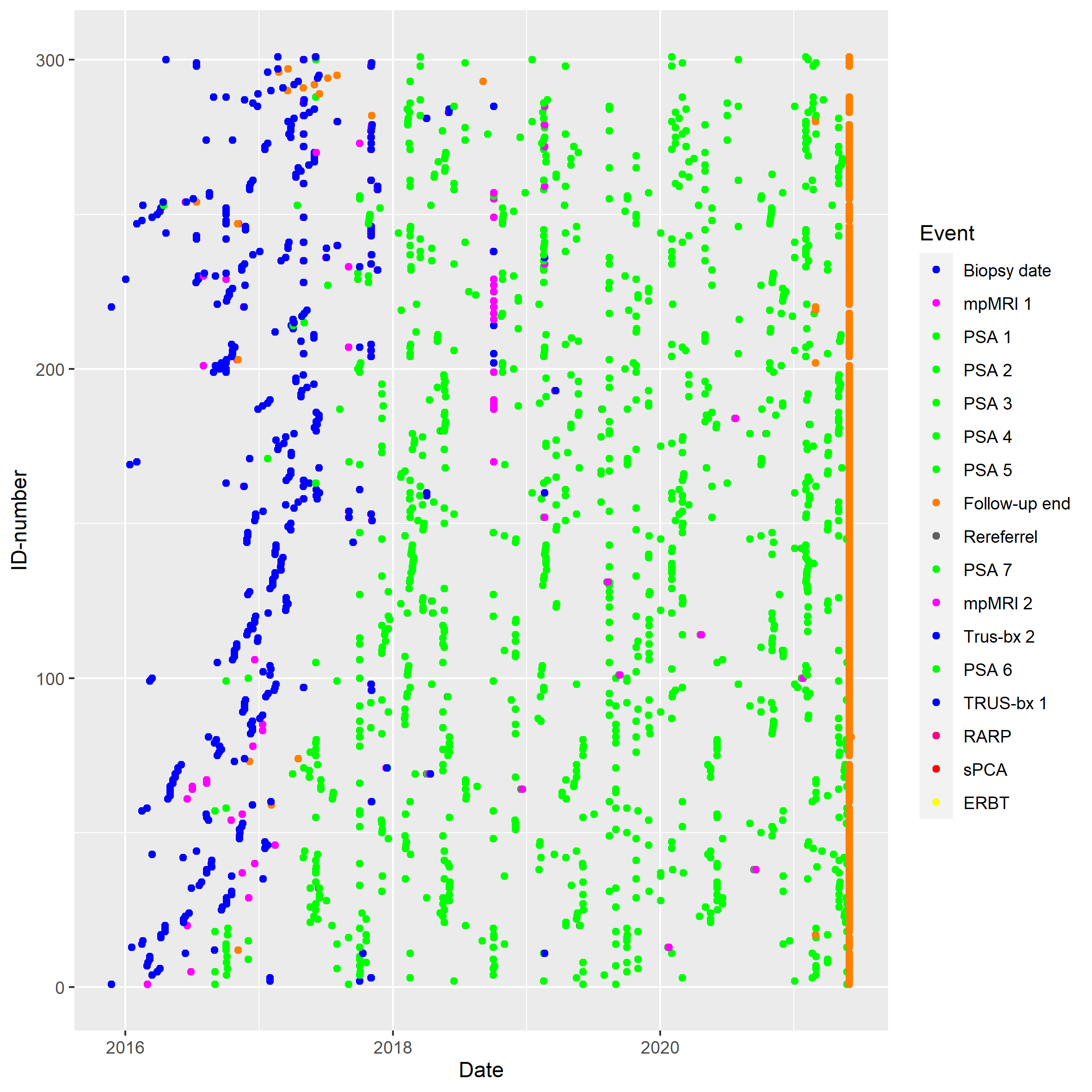


Abbreviations: PSA, prostate-specific-antigen; mpMRI, multiparametric magnetic resonance imaging; Trus-bx, transrectal ultrasound-guided; RARP, radical prostatectomy; ERBT, external beam radiation therapy; sPCA, significant prostate cancer.
