# Supplementary material for: Outcome of 5-year follow-up in men with negative findings on initial biparametric MRI
Source: Heliyon. 2021 Nov 6;7(11):e08325. doi: 10.1016/j.heliyon.2021.e08325 (PMC8601994; doi:10.1016/j.heliyon.2021.e08325)
Supplement: sup. Table 1 [file mmc3.docx]

**Table 1: Detailed descriptions of the mpMRI and bpMRI protocols**

***Example with a man of 83 kg***

***T2 weighted sagital:***

FOV AP (mm) = 160;

FH (mm) = 197.647064;

RL (mm) = 78.9000015;

ACQ voxel size AP (mm) = 0.600000024;

FH (mm) = 0.5744403;

Slice thickness (mm) = 3;

Recon voxel size AP (mm) = 0.294117659;

FH (mm) = 0.294117659;

Fold-over suppression = "oversampling";

H (mm) = 100.780945;

F (mm) = 100.780945;

Reconstruction matrix = 672;

SENSE = "no";

k-t Acceleration = "no";

Stacks = 1;

type = "parallel";

slices = 24;

slice gap = "default";

slice orientation = "sagittal";

fold-over direction = "FH";

fat shift direction = "P";

Stack Offc. AP (P=+mm) = 25.8316669;

RL (L=+mm) = 5.41084766;

FH (H=+mm) = 26.0951462;

Ang. AP (deg) = 0;

RL (deg) = -0;

FH (deg) = -0;

Free rotatable = "no";

Minimum number of packages = 1;

Slice scan order = "default";

Large table movement = "no";

PlanAlign = "no";

REST slabs = 0;

Interactive positioning = "no";

Patient position = "feet first";

Patient body position = "feet first";

Patient orientation = "supine";

Patient body orientation = "supine";

Scan type = "Imaging";

Scan mode = "MS";

technique = "SE";

Modified SE = "no";

Acquisition mode = "cartesian";

Fast Imaging mode = "TSE";

shot mode = "multishot";

TSE factor = 22;

TE spacing = "user defined";

(ms) = 9;

startup echoes = 0;

profile order = "asymmetric";

DRIVE = "yes";

ultrashort = "no";

fid reduction = "default";

Echoes = 1;

partial echo = "no";

TE = "user defined";

(ms) = 90;

Flip angle (deg) = 90;

Refocusing control = "constant";

angle (deg) = 100;

TR = "range";

minimum (ms) = 3000;

maximum (ms) = 8000;

Halfscan = "no";

Water-fat shift = "minimum";

RF Shims = "adaptive";

Shim = "default";

mDIXON = "no";

Fat suppression = "no";

Water suppression = "no";

MTC = "no";

Diffusion mode = "no";

SAR mode = "high";

B1 mode = "default";

SAR allow first level = "yes";

Patient WB SAR [W/kg] = 0;

Patient Head SAR [W/kg] = 0;

Patient max. dB/dt [T/s] = 0;

Max slewrate [T/m/s] = 0;

Max. B1+rms [uT] = 0;

PNS mode = "low";

Gradient mode = "default";

SofTone mode = "no";

Cardiac synchronization = "no";

Respiratory compensation = "no";

Navigator respiratory comp = "no";

Flow compensation = "yes";

direction = "in-plane";

Temporal slice spacing = "default";

Motion smoothing = "yes";

NSA = 2;

SMART = "no";

Manual start = "no";

Dynamic study = "no";

Arterial Spin labeling = "no";

Preparation phases = "auto";

Interactive F0 = "no";

Quick Survey = "default";

MIP/MPR = "no";

Images = "M", (3) "no";

Autoview image = "M";

Calculated images = (4) "no";

Reference tissue = "Skeletal muscle";

Recon compression = "No";

Preset window contrast = "intermediate";

Reconstruction mode = "real time";

Save raw data = "no";

Hardcopy protocol = "no";

Image filter = "system default";

Uniformity correction = "no";

Geometry correction = "default";

IF_info_seperator = 1634755923;

Total scan duration = "06:17.8";

Rel. SNR = 1;

Act. TR (ms) = "3096";

Act. TE (ms) = "90";

ACQ matrix M x P = "268 x 326";

ACQ voxel MPS (mm) = "0.60 / 0.61 / 3.00";

REC voxel MPS (mm) = "0.29 / 0.29 / 3.00";

Scan percentage (%) = 98.6547089;

Packages = 2;

Min. slice gap (mm) = 3;

Act. slice gap (mm) = 0.300000012;

WFS (pix) / BW (Hz) = "0.838 / 518.2";

Full flow comp. = "yes";

TSE es / shot (ms) = "9.0 / 207";

TEeff / TEequiv (ms) = "90 / 73 ";

Min. TR (ms) = "3096";

Local torso SAR = "< 100 %";

Whole body SAR / level = "< 2.7 W/kg / 1st level";

SED = "< 1.0 kJ/kg";

Coil Power = "80 %";

Max B1+rms = "2.09 uT";

PNS / level = "59 % / normal";

dB/dt = "34.1 T/s";

Sound Pressure Level (dB) = 23.3350964;

***T2 weighted axial:***

FOV AP (mm) = 180;

RL (mm) = 180;

FH (mm) = 102;

ACQ voxel size AP (mm) = 0.449999988;

RL (mm) = 0.448877811;

Slice thickness (mm) = 3;

Recon voxel size AP (mm) = 0.224999994;

RL (mm) = 0.224999994;

Fold-over suppression = "oversampling";

L (mm) = 180;

R (mm) = 180;

Reconstruction matrix = 800;

SENSE = "no";

k-t Acceleration = "no";

Stacks = 1;

type = "parallel";

slices = 34;

slice gap = "user defined";

gap (mm) = 0;

slice orientation = "transverse";

fold-over direction = "RL";

fat shift direction = "P";

Stack Offc. AP (P=+mm) = 14.0835333;

RL (L=+mm) = -6.31259155;

FH (H=+mm) = -15.2730064;

Ang. AP (deg) = -1.55300523e-18;

RL (deg) = -2.03658628;

FH (deg) = 1.68857598;

Free rotatable = "no";

Minimum number of packages = 1;

Slice scan order = "default";

Large table movement = "no";

PlanAlign = "no";

REST slabs = 0;

Interactive positioning = "no";

Patient position = "feet first";

Patient body position = "feet first";

Patient orientation = "supine";

Patient body orientation = "supine";

Scan type = "Imaging";

Scan mode = "MS";

technique = "SE";

Modified SE = "no";

Acquisition mode = "cartesian";

Fast Imaging mode = "TSE";

shot mode = "multishot";

TSE factor = 17;

startup echoes = 0;

profile order = "linear";

DRIVE = "no";

ultrashort = "no";

fid reduction = "default";

Echoes = 1;

partial echo = "no";

TE = "user defined";

(ms) = 90;

Flip angle (deg) = 90;

Refocusing control = "constant";

angle (deg) = 120;

TR = "range";

minimum (ms) = 3000;

maximum (ms) = 8000;

Halfscan = "no";

Water-fat shift = "user defined";

(pixels) = 2;

RF Shims = "adaptive";

Shim = "default";

mDIXON = "no";

Fat suppression = "no";

Water suppression = "no";

MTC = "no";

Diffusion mode = "no";

SAR mode = "high";

B1 mode = "default";

SAR allow first level = "yes";

Patient pregnancy = "no";

Patient WB SAR [W/kg] = 0;

Patient Head SAR [W/kg] = 0;

Patient max. dB/dt [T/s] = 0;

Max slewrate [T/m/s] = 0;

Max. B1+rms [uT] = 0;

PNS mode = "high";

Gradient mode = "default";

SofTone mode = "no";

Cardiac synchronization = "no";

Respiratory compensation = "no";

Navigator respiratory comp = "no";

Flow compensation = "no";

Temporal slice spacing = "default";

Motion smoothing = "yes";

NSA = 1;

Manual start = "no";

Dynamic study = "no";

Arterial Spin labeling = "no";

Preparation phases = "auto";

Interactive F0 = "no";

Quick Survey = "default";

MIP/MPR = "no";

Images = "M", (3) "no";

Autoview image = "M";

Calculated images = (4) "no";

Reference tissue = "Liver";

Recon compression = "No";

Preset window contrast = "soft";

Reconstruction mode = "immediate";

Save raw data = "no";

Hardcopy protocol = "no";

Image filter = "system default";

Uniformity correction = "no";

Geometry correction = "default";

IF_info_seperator = 1634755923;

Total scan duration = "09:19.3";

Rel. SNR = 1;

Act. TR (ms) = "3938";

Act. TE (ms) = "90";

ACQ matrix M x P = "400 x 396";

ACQ voxel MPS (mm) = "0.45 / 0.45 / 3.00";

REC voxel MPS (mm) = "0.22 / 0.22 / 3.00";

Scan percentage (%) = 99.0840988;

Packages = 2;

Min. slice gap (mm) = 3;

WFS (pix) / BW (Hz) = "1.988 / 218.5";

TSE es / shot (ms) = "10.0 / 170";

TEeff / TEequiv (ms) = "90 / 79 ";

Min. TR (ms) = "3938";

Local torso SAR = "< 100 %";

Whole body SAR / level = "< 2.7 W/kg / 1st level";

SED = "< 1.5 kJ/kg";

Coil Power = "80 %";

Max B1+rms = "2.09 uT";

PNS / level = "48 % / normal";

dB/dt = "37.6 T/s";

Sound Pressure Level (dB) = 18.9890804;

***T2 weighted coronal:***

FOV FH (mm) = 190;

RL (mm) = 190;

AP (mm) = 79;

ACQ voxel size FH (mm) = 0.600000024;

RL (mm) = 0.600203454;

Slice thickness (mm) = 3;

Recon voxel size FH (mm) = 0.219907403;

RL (mm) = 0.219907403;

Fold-over suppression = "oversampling";

L (mm) = 200;

R (mm) = 200;

Reconstruction matrix = 864;

SENSE = "yes";

P reduction (RL) = 1.5;

k-t Acceleration = "no";

Stacks = 1;

type = "parallel";

slices = 20;

slice gap = "user defined";

gap (mm) = 1;

slice orientation = "coronal";

fold-over direction = "RL";

fat shift direction = "F";

Stack Offc. AP (P=+mm) = 17.9099503;

RL (L=+mm) = 5.41084766;

FH (H=+mm) = 39.1660042;

Ang. AP (deg) = 0;

RL (deg) = -9.32226753;

FH (deg) = -0;

Free rotatable = "no";

Minimum number of packages = 1;

Slice scan order = "default";

Large table movement = "no";

PlanAlign = "no";

REST slabs = 0;

Interactive positioning = "no";

Patient position = "feet first";

Patient body position = "feet first";

Patient orientation = "supine";

Patient body orientation = "supine";

Scan type = "Imaging";

Scan mode = "MS";

technique = "SE";

Modified SE = "no";

Acquisition mode = "cartesian";

Fast Imaging mode = "TSE";

shot mode = "multishot";

TSE factor = 9;

startup echoes = 0;

profile order = "linear";

DRIVE = "no";

ultrashort = "no";

fid reduction = "default";

Echoes = 1;

partial echo = "no";

TE = "user defined";

(ms) = 90;

Flip angle (deg) = 90;

Refocusing control = "constant";

angle (deg) = 140;

TR = "range";

minimum (ms) = 3000;

maximum (ms) = 8000;

Halfscan = "no";

Water-fat shift = "user defined";

(pixels) = 2;

RF Shims = "adaptive";

Shim = "default";

mDIXON = "no";

Fat suppression = "no";

Water suppression = "no";

MTC = "no";

Diffusion mode = "no";

SAR mode = "high";

B1 mode = "user defined";

amplitude (uT) = 10;

SAR allow first level = "yes";

Patient pregnancy = "no";

Patient WB SAR [W/kg] = 0;

Patient Head SAR [W/kg] = 0;

Patient max. dB/dt [T/s] = 0;

Max slewrate [T/m/s] = 0;

Max. B1+rms [uT] = 0;

PNS mode = "low";

Gradient mode = "default";

SofTone mode = "no";

Cardiac synchronization = "no";

Respiratory compensation = "no";

Navigator respiratory comp = "no";

Flow compensation = "no";

Temporal slice spacing = "default";

Motion smoothing = "yes";

NSA = 1;

Manual start = "no";

Dynamic study = "no";

Arterial Spin labeling = "no";

Preparation phases = "auto";

Interactive F0 = "no";

Quick Survey = "default";

MIP/MPR = "no";

Images = "M", (3) "no";

Autoview image = "M";

Calculated images = (4) "no";

Reference tissue = "Liver";

Recon compression = "No";

Preset window contrast = "soft";

Reconstruction mode = "real time";

Save raw data = "no";

Hardcopy protocol = "no";

Image filter = "system default";

Uniformity correction = "no";

Geometry correction = "default";

IF_info_seperator = 1634755923;

Total scan duration = "04:15.8";

Rel. SNR = 1;

Act. TR (ms) = "3504";

Act. TE (ms) = "90";

ACQ matrix M x P = "316 x 313";

ACQ voxel MPS (mm) = "0.60 / 0.61 / 3.00";

REC voxel MPS (mm) = "0.22 / 0.22 / 3.00";

Scan percentage (%) = 98.9312973;

Packages = 1;

Min. slice gap (mm) = -0;

WFS (pix) / BW (Hz) = "1.999 / 217.3";

TSE es / shot (ms) = "18.0 / 162";

TEeff / TEequiv (ms) = "90 / 84 ";

Min. TR (ms) = "3504";

Local torso SAR = "< 83 %";

Whole body SAR / level = "< 2.2 W/kg / 1st level";

SED = "< 0.6 kJ/kg";

Coil Power = "66 %";

Max B1+rms = "1.91 uT";

PNS / level = "42 % / normal";

dB/dt = "36.9 T/s";

Sound Pressure Level (dB) = 15.241437;

***DWI B-value = 2000***

FOV AP (mm) = 180;

RL (mm) = 180;

FH (mm) = 102;

ACQ voxel size AP (mm) = 2.20000005;

RL (mm) = 2.20223308;

Slice thickness (mm) = 3;

Recon voxel size AP (mm) = 0.803571403;

RL (mm) = 0.803571403;

Fold-over suppression = "oversampling";

L (mm) = 110;

R (mm) = 110.806442;

Reconstruction matrix = 224;

SENSE = "yes";

P reduction (RL) = 2;

k-t Acceleration = "no";

Stacks = 1;

type = "parallel";

slices = 34;

slice gap = "user defined";

gap (mm) = 0;

slice orientation = "transverse";

fold-over direction = "RL";

fat shift direction = "L";

Stack Offc. AP (P=+mm) = 18.1115284;

RL (L=+mm) = 16.2324886;

FH (H=+mm) = 11.6415367;

Ang. AP (deg) = 0;

RL (deg) = -1.99996734;

FH (deg) = -0;

Free rotatable = "no";

Minimum number of packages = 1;

Slice scan order = "interleaved";

Large table movement = "no";

PlanAlign = "no";

REST slabs = 0;

Interactive positioning = "no";

Patient position = "feet first";

Patient body position = "feet first";

Patient orientation = "supine";

Patient body orientation = "supine";

Scan type = "Imaging";

Scan mode = "MS";

technique = "SE";

Modified SE = "no";

Acquisition mode = "cartesian";

Fast Imaging mode = "EPI";

shot mode = "single-shot";

Echoes = 1;

partial echo = "no";

TE = "shortest";

Flip angle (deg) = 90;

TR = "shortest";

Halfscan = "yes";

factor = 0.698113024;

Water-fat shift = "minimum";

RF Shims = "adaptive";

Shim = "auto";

mDIXON = "no";

Fat suppression = "SPAIR";

power = "1";

inversion delay = "auto";

suppr. level = "strong";

frequency offset = "default";

Grad Rev Fat suppr = "yes";

Water suppression = "no";

MTC = "no";

Diffusion mode = "DWI";

gradient overplus = "yes";

nr of b-factors = 4;

b-factor order = "user defined";

b-factors = 100, 800, 2000,

(1021) 0;

average high b = "yes";

SAR mode = "high";

B1 mode = "default";

SAR allow first level = "yes";

Patient pregnancy = "no";

Patient WB SAR [W/kg] = 0;

Patient Head SAR [W/kg] = 0;

Patient max. dB/dt [T/s] = 0;

Max slewrate [T/m/s] = 0;

Max. B1+rms [uT] = 0;

PNS mode = "high";

Gradient mode = "enhanced";

SofTone mode = "no";

Cardiac synchronization = "no";

Respiratory compensation = "no";

Navigator respiratory comp = "no";

Flow compensation = "no";

Temporal slice spacing = "default";

NSA = 2;

SMART = "no";

Manual start = "no";

Dynamic study = "no";

Arterial Spin labeling = "no";

Preparation phases = "auto";

Interactive F0 = "no";

Quick Survey = "default";

MIP/MPR = "no";

Images = "M", (3) "no";

Autoview image = "M";

Calculated images = (4) "no";

Reference tissue = "Liver";

Recon compression = "No";

Preset window contrast = "soft";

Reconstruction mode = "immediate";

Save raw data = "no";

Hardcopy protocol = "no";

Image filter = "system default";

Uniformity correction = "no";

Geometry correction = "default";

IF_info_seperator = 1634755923;

Total scan duration = "08:54.7";

Rel. SNR = 3.3532536;

Act. TR (ms) = "13710";

Act. TE (ms) = "72";

ACQ matrix M x P = "84 x 80";

ACQ voxel MPS (mm) = "2.14 / 2.24 / 3.00";

REC voxel MPS (mm) = "0.80 / 0.80 / 3.00";

Scan percentage (%) = 95.7894745;

Packages = 1;

Min. slice gap (mm) = -0;

EPI factor = 91;

WFS (pix) / BW (Hz) = "29.181 / 14.9";

BW in EPI freq. dir. (Hz) = "2779.1";

Local torso SAR = "< 23 %";

Whole body SAR / level = "< 0.6 W/kg / normal";

SED = "< 0.3 kJ/kg";

Coil Power = "19 %";

Max B1+rms = "1.01 uT";

PNS / level = "87 % / 1st level";

dB/dt = "53.4 T/s";

Sound Pressure Level (dB) = 19.8596325;

***Dynamic contrast-enhanced T1weighted***

Patient weight [kg] = 83;

SmartSelect = "yes";

Coil 1 (exclude) = "None";

Uniformity = "CLEAR";

FOV RL (mm) = 180;

AP (mm) = 157.5;

FH (mm) = 104;

ACQ voxel size RL (mm) = 0.699999988;

AP (mm) = 0.711805522;

FH (mm) = 10.3999996;

Recon voxel size RL (mm) = 0.703125;

AP (mm) = 0.703125;

FH (mm) = 5.19999981;

Fold-over suppression = "oversampling";

P (mm) = 75;

A (mm) = 75;

Slice oversampling = "default";

ENCASE enable = "no";

Reconstruction matrix = 256;

SENSE = "yes";

P reduction (AP) = 2.20000005;

S reduction (FH) = 1;

k-t Acceleration = "no";

Stacks = 1;

slices = 20;

slice orientation = "transverse";

fold-over direction = "AP";

fat shift direction = "L";

Stack Offc. AP (P=+mm) = 17.9099503;

RL (L=+mm) = 5.41084766;

FH (H=+mm) = 39.1660042;

Ang. AP (deg) = 0;

RL (deg) = -9.32226753;

FH (deg) = -0;

Free rotatable = "no";

Multi-chunk = "no";

Large table movement = "no";

PlanAlign = "no";

REST slabs = 2;

shared = "no";

type = (12) "parallel";

circular slabs = (12) 10;

orientation = (12) "transverse";

thickness (mm) = (12) 60;

position = "feet", "head",

(10) "left";

gap = (12) "default";

(mm) = (2) 15, (10) 20;

distance (mm) = (12) 50;

Rest Offc. AP (P=+mm) = 2.19717813,

33.6227226, (10) 0;

RL (L=+mm) = (2) 5.41084766,

(10) 0;

FH (H=+mm) = -56.5529022,

134.884903, (10) 0;

Ang. AP (deg) = (12) 0;

RL (deg) = (2) -9.32226753,

(10) 0;

FH (deg) = (12) -0;

angulation (deg) = (12) 0;

FM shape = (12) "no";

composite elements = (12) 1;

correction factor = (12) 1;

power = (12) "1";

target = (12) "fat";

timed = "no";

start (phase) = 1;

stop (phase) = 2;

delay = "no";

(ms) = 50;

Interactive positioning = "no";

Patient position = "feet first";

Patient body position = "feet first";

Patient orientation = "supine";

Patient body orientation = "supine";

Scan type = "Imaging";

Scan mode = "3D";

technique = "FFE";

Contrast enhancement = "T1";

Acquisition mode = "cartesian";

Fast Imaging mode = "TFE";

shot mode = "multishot";

TFE factor = 50;

startup echoes = "user defined";

(number) = 0;

shot interval = "shortest";

profile order = "low_high";

turbo direction = "radial";

Echoes = 1;

partial echo = "no";

shifted echo = "no";

TE = "shortest";

Flip angle (deg) = 12;

TR = "shortest";

Halfscan = "yes";

factor Y = 0.625;

factor Z = 1;

Water-fat shift = "maximum";

RF Shims = "adaptive";

Shim = "auto";

mDIXON = "no";

Fat suppression = "SPAIR";

power = "1";

inversion delay = "auto";

suppr. level = "strong";

frequency offset = "default";

Water suppression = "no";

TFE prepulse = "no";

MTC = "no";

T2prep = "no";

Diffusion mode = "no";

SAR mode = "high";

B1 mode = "user defined";

amplitude (uT) = 10;

SAR allow first level = "yes";

Patient pregnancy = "no";

Patient WB SAR [W/kg] = 0;

Patient Head SAR [W/kg] = 0;

Patient max. dB/dt [T/s] = 0;

Max slewrate [T/m/s] = 0;

Max. B1+rms [uT] = 0;

PNS mode = "moderate";

Gradient mode = "default";

SofTone mode = "no";

Cardiac synchronization = "no";

Respiratory compensation = "no";

Navigator respiratory comp = "no";

Flow compensation = "no";

fMRI echo stabilisation = "no";

NSA = 1;

Angio / Contrast enh. = "no";

Quantitative flow = "no";

CENTRA = "no";

Manual start = "yes";

Dynamic study = "individual";

dyn scans = 12;

dyn scan times = "shortest";

dummy scans = 0;

immediate subtraction = "no";

fast next scan = "no";

synch. ext. device = "no";

dyn stabilization = "no";

prospect. motion corr. = "no";

Keyhole = "no";

Arterial Spin labeling = "no";

Preparation phases = "auto";

Interactive F0 = "no";

Quick Survey = "default";

MIP/MPR = "no";

SWIp = "no";

Images = "M", (3) "no";

Autoview image = "M";

Calculated images = (4) "no";

Reference tissue = "Liver";

Recon compression = "No";

Preset window contrast = "soft";

Reconstruction mode = "real time";

Save raw data = "no";

Hardcopy protocol = "no";

Image filter = "system default";

Uniformity correction = "no";

Geometry correction = "default";

Elliptical k-space shutter = "default";

IF_info_seperator = 1634755923;

Total scan duration = "03:03.9";

Rel. SNR = 1;

Act. TR/TE (ms) = "10 / 5.0";

Dyn. scan time = "00:15.2";

ACQ matrix M x P = "256 x 222";

ACQ voxel MPS (mm) = "0.70 / 0.71 / 10.4";

REC voxel MPS (mm) = "0.70 / 0.70 / 5.20";

Scan percentage (%) = 99.0049744;

Act. slice gap (mm) = -5.19999981;

TFE shots = 23;

TFE dur. shot / acq (ms) = "661.6 / 513.7";

TFE shot interval (ms) = 661.557373;

Act. WFS (pix) / BW (Hz) = "3.229 / 134.5";

Min. WFS (pix) / Max. BW (Hz) = "0.694 / 626.0";

Local torso SAR = "< 26 %";

Whole body SAR / level = "< 0.7 W/kg / normal";

SED = "< 0.1 kJ/kg";

Coil Power = "21 %";

Max B1+rms = "1.08 uT";

PNS / level = "64 % / normal";

dB/dt = "50.9 T/s";

Sound Pressure Level (dB) = 18.3472195;

*Abbreviations: TR, repetition time; TE, echo time; FOV, field of view; NSA, number of signal averages; Temp res, temporal resolution; sag, sagittal; ax, axial; DWI, diffusion-weighted images; DCE, dynamic contrast-enhanced; ms, milliseconds.*
